# Effects of a novel low volume resuscitation solutions on coagulation and platelet function

**DOI:** 10.1371/journal.pone.0215386

**Published:** 2019-05-01

**Authors:** Loren K. Liebrecht, Jason Newton, Erika J. Martin, Niluka Wickramaratne, Sudha Jayaraman, Jinfeng Han, Michel Aboutanos, Donald F. Brophy, Martin J. Mangino

**Affiliations:** 1 Department of Surgery, Division of Acute Care Surgery, Virginia Commonwealth University School of Medicine, Richmond, VA, United States of America; 2 Department of Biochemistry and Molecular Biology, Virginia Commonwealth University School of Medicine, Richmond, VA, United States of America; 3 Department of Pharmacotherapy and Outcomes Science, Virginia Commonwealth University School of Pharmacy, Richmond, VA, United States of America; 4 Department of Physiology and Biophysics, Virginia Commonwealth University School of Medicine, Richmond, VA, United States of America; 5 Department of Emergency Medicine, Virginia Commonwealth University School of Medicine, Richmond, VA, United States of America; University of South Carolina, UNITED STATES

## Abstract

**Background:**

Novel crystalloid solutions containing polyethylene glycol polymers (PEG-20k) produce dramatic resuscitation effects but dose-dependently produce a hypocoagulative state. The objective of this study was to examine possible mechanisms of this effect. Based on previous thromboelastography data, we hypothesize the effect is largely due to platelet interactions with the polymers.

**Methods:**

Whole citrated blood from healthy volunteers was diluted ex-vivo 10% with crystalloids and tested for coagulation and platelet function. The specific tests included prothrombin time (PT), activated partial thromboplastin time (aPTT), fibrinogen and von Willebrand factor (vWf) activity, thrombin generation, thromboelastography with and without platelet mapping, platelet flow cytometry, and erythrocyte sedimentation rate.

**Findings:**

Fibrinogen and vWF activities, PT, and aPTT were not affected by PEG-20k dilutions. Thrombin activity was mildly suppressed with PEG-20k (TTP- 20%). Platelet mapping demonstrated significantly greater % inhibition of both ADP and arachidonic acid-induced platelet aggregation with PEG-20k, but direct ADP-activated gpIIa/IIIb (PAC1) and P-selectin (CD62P) binding site expression was not altered. Mild dose-dependent suppression of TEG-MA was seen with PEG-20k using platelet poor plasma. Erythrocyte Sedimentation Rates (ESR) were dramatically accelerated after dilution with 10% PEG-20k, which was competitively blocked by smaller PEG polymers, suggesting nonspecific PEG-20k cell binding effects.

**Conclusions:**

PEG-20k creates a mild hypocoagulative state in whole blood at concentrations ≥10%, which may be due to platelet-PEG interactions at the IIb/IIIa interface with lesser effects on fibrin polymerization. This interaction may cause a functional thrombasthenia induced by nonspecific platelet surface passivation by the PEG polymer.

## Introduction

Trauma is the number one cause of death for people under 44 years of age in the US and the third leading cause of death overall for all age groups. Trauma accounts for about 30% of all life-years lost in the US, compared to cancer (16%), heart disease (12%), and HIV (2%) [1]. For all traumatic injuries, hemorrhagic shock is responsible for over 35% of pre-hospital deaths and over 40% of all deaths within the first 24 hours. This is second only to deaths induced by severe CNS injury [2]. Hemorrhagic hypotension exposes the patient to immediate complications of life-threatening infections, coagulopathies, and multiple organ failure [3, 4].

Crystalloid-based intravenous (IV) solutions are available for pre-hospital use because they can be safely transported and stored but they are generally limited in their effectiveness. Only a fraction of infused crystalloid volume stays in the intravascular space and the use of low volume crystalloids has minimal effects on pressure and perfusion [5, 6]. The movement of crystalloid fluid from capillary to interstitium is compounded by the increase in capillary permeability from trauma-related inflammation and trauma-induced capillary leak syndrome (TICS) [7]. Furthermore, crystalloid resuscitation exacerbates TICS, acidosis, hypothermia, and coagulopathy [7, 8]. Other resuscitation solutions such as hypertonic saline or starch have had disappointing results [9, 10] including concerns and risks associated with their use [8, 11]. There remains a need for a better crystalloid fluid that can be given at a low volume to resuscitate patients in severe hemorrhagic shock awaiting definitive treatment, especially for the prehospital setting.

Recently, polyethylene glycol (PEG) polymers of specific molecular weight ranges have been used in crystalloid solutions to act as highly effective low-volume resuscitation (LVR) solutions [6, 12–14]. These polymers non-energetically move isotonic fluid from intracellular and interstitial spaces into the capillary space by simple osmotic actions in response to metabolic cell swelling that occurs in shocked and ischemic tissues. As water flow moves from the interstitial spaces to the capillaries, the capillary exchange in the tissues dramatically improves under very low volume conditions because the microcirculation is decompressed while the capillary spaces are re-loaded with volume and pressure for driving flow [14]. This causes rapid clearance of lactate, increased blood pressure, and tolerance to the low volume state [12]. While these polymers work several-fold better than hydroxyethyl starch based polymers [6, 13, 14], implying different mechanisms of action, interference with blood clotting and coagulation may be shared by both types of polymers. For example, the I.V. starch-based crystalloid solutions Hextend and Hespan are complicated by both renal toxicity and coagulopathies [15], which in trauma settings are a concern.

In a set of experiments recently published [16], we described detailed thromboelastography (TEG) evidence of a mild hypocoagulative state induced by 10% dilutions of blood samples from healthy volunteers and from blood samples from trauma patients with 10% PEG-20,000 Da (PEG-20k) solutions. The TEG-based data suggested PEG-20k had effects on not only final clot strength (maximal amplitude, MA), but also on the clot propagation parameters *k* and α-angle, which are measurements influenced by fibrinogen cross-linking. The PEG-20k effects on TEG parameters were significantly different, relative to those of normal saline and hetastarch, and appeared in a dose-dependent fashion.

Therefore, the aim of this study was to characterize the mechanism of the dose-dependent hypocoagulopathy findings in the TEG parameters observed with PEG-20k solutions. To that end, we systematically studied a battery of coagulation and platelet function parameters in blood samples obtained from healthy volunteers diluted 10% with PEG-20k solution. From our previous TEG analysis, we hypothesize that the hypocoagulable state induced by PEG-20k solutions on whole clotting blood is related mainly to interferences of the polymer with platelet function.

## Methods

### Volunteer blood

This study was done under the approval of the VCU Institutional Review Board (approval number HM20002817). Each blood donor for this study provided written consent. Whole blood (15-ml) was drawn into citrated vacutainer collection tubes from 12 healthy consented volunteers (18–50 years of age) of both sexes that were free of all medications and tobacco. All volunteers donated blood under a VCU approved IRB protocol. The blood was diluted 10% in the lab with solutions of 10%, 7.5%, and 5% PEG-20k in saline (0.9% NaCl), 6% Hextend, or a 0.9% NaCl solution that served always as a paired dilutional control for all test solutions. Immediately after the dilutions, the samples were analyzed for coagulation parameters. One mL of citrated whole blood aliquots was used for TEG analysis using kaolin as activator, and the remaining citrated whole blood was centrifuged at 180 x g for 10 minutes to obtain platelet rich plasma (PRP). Platelet poor plasma (PPP) was obtained by double centrifugation of the remaining plasma at 2000 x g for 10 min at room temperature. PRP was then diluted with autologous PPP to yield a final platelet count of 150 x10^9^/L for platelet-dependent thrombin generation assays. The remaining PPP was used for the analysis of platelet-independent thrombin generation, PT, aPTT, fibrinogen, and vWF concentrations. Platelet counts were performed with an automated cell counter (ABX Micros 60, Horiba Medical, Irvine, CA, USA). The time between blood draw and analysis was less than two hours. Normal values have been previously described [17–20].

### Fibrinogen, PT, aPTT, vWF

Fibrinogen, PT, aPTT, and von Willebrand factor antigen (vWF) function were measured in plasma using standard assays (STA fibrinogen clotting activity assay, PT-Neoplastin CI, PTT- Automate, PTT CK Prest, and Liatest vWF assays, respectively) on the STA Compact analyzer (Diagnostica Stago, Parsippany, NJ, USA) according to manufacturer’s instructions.

### Thrombin generation assay

The kinetics of thrombin generation was assessed in PRP and in PPP according to methods previously described by Hemker, et al [19]. Briefly, 20 μl of trigger reagent (1pM Tissue Factor), and 80 μl PPP were manually pipetted in triplicate into 96-well microtiter plates (Immulon 2HB plate; Diagnostica Stago, Parsippany, NJ, USA). The plate was placed in the fluorometer for a 10 minute 37°C incubation (Fluoroskan AscentTM; Thermolab Systems OY, Helsinki, Finland). The device was equipped with a 390/460 filter set. Twenty μl of starting reagent containing the fluorogenic substrate Z-GGR-AMC (2.5 mM) and CaCl2 (100 mM) were automatically dispensed into each well immediately before measurement initiation. Thrombin generation curves were calculated using the calibrated automated thrombogram (Thrombinoscope BV, Masstricht, The Netherlands) software version: V5.0.0.742. The thrombogram parameters (lag time, peak thrombin concentration, and endogenous thrombin potential (ETP), which reflected the maximum amount of thrombin that a sample could potentially generate) were reported.

### TEG and platelet mapping

Thromboelastography with platelet mapping was determined using a TEG 5000 (Haemonetics Corp., Braintree, Mass) using the intrinsic pathway activator kaolin (Haemonetics Corp.) and recalcification to 10 mmol/L final calcium concentration. The TEG 5000 reported time to onset of clot formation (R), which positively correlates with thrombin generation; the time to reach a predetermined level of clot stiffness (K) and the clotting angle (α-angle), which correlates with fibrin polymerization; the maximal amplitude (MA) or stiffness, representing clot strength. Platelet mapping was done using the TEG-5000 instrument and a platelet mapping kit (Haemonetics Corp.) that tests the platelet component to clot formation (MA) on TEG [21]. Briefly, heparinized blood treated with reptilase and activated factor XIII was used to form thrombin independent cross-linked fibrin. The platelet specific component for the adenosine diphosphate (ADP) and thromboxane receptor pathways were determined by activation with ADP and arachidonic acid (AA), respectively, on the heparinized blood samples. All assays were performed according to manufacturer guidelines.

### TEG on platelet poor plasma (PPP)

To test the effects of PEG-20k on just the fibrin component of clot formation, TEG was performed on platelet poor plasma. Citrated whole blood was drawn from six healthy volunteers as before and the plasma was obtained by centrifugation at 2500 x g for 10 minutes. The PPP was diluted 10% with solutions of PEG-20k at 0%, 5%, 10%, and 15%. Standard TEG using kaolin activation was performed as before on each sample immediately after PEG-20k dilution and compared to the 10% dilutional volume control (0% PEG-20k) using saline.

### Platelet flow cytometry

Platelet activation was also quantified using flow cytometric analysis. Briefly, flow cytometry was performed on a BD Biosciences device (BD Biosciences Accuri™ C6 Flow Cytometer, San Jose, CA, USA) using citrated whole blood according to current standards from the European Working Group on Cell Analysis [22]. CD41a conjugated with PE-Cy5 (Mouse Anti-Human, BD Pharmingen, Franklin Lakes, NJ, USA), PAC-1 conjugated with FITC (BD Biosciences), and CD62p conjugated with PE (Mouse Anti-Human, BD Pharmingen) were used to identify platelets and to identify their activation status. Corresponding isotypic-matched monoclonal antibodies PE-Mouse IgG1-K isotype, FITC-Mouse IgM-K isotype and PE-Cy5-Mouse IgG1-K isotype (BD Pharmingen) were used as negative controls. A portion of the whole blood specimens was treated with 0.005 mL of ADP (3 μM final) for platelet activation. Samples were analyzed under the following conditions: Fluidics: medium; Forward scatter threshold: 30,000; and 20,000 events were collected in a preset platelet gate using standard methods including CD41a as a global platelet marker. Results are expressed in mean fluorescence intensity units for CD41 and in percentages for other markers of activation. Flow cytometry measured platelet activation via the glycoprotein P-selectin, because it rapidly translocates to the platelet surface on stimulation. The P-selectin content on the platelet surface was detected with the CD62-P mAb. Also measured was the glycoprotein IIb/IIIa surface integrin transition to its high-affinity state by using the mAb against high-affinity glycoprotein IIb/IIIa platelet surface integrin (PAC-1) conjugated with fluorescein isothiocyanate (BD Biosciences).

### ESR

The erythrocyte sedimentation rate was measured in diluted citrated whole blood using the Sediplast Westergren ESR system tests (Polymedco, Inc., Cortiandt Manor, NY). About 1 ml of whole blood was drawn up into a 10 cm Westergren ESR tube, which was held in the vertical position for 75 minutes. The rate of red blood cell sedimentation was measured as the migration (in mm) of the red cell column down the tube under the force of gravity. Blood samples diluted with 10% volume of saline (volume control) were compared to 10% dilution with PEG-20k solutions and PEG-20k solutions with other test compounds.

### Statistical data analysis

All statistical analysis was performed using GraphPad Prism version 6.07 for Windows (GraphPad Software, La Jolla California USA). Data groups were analyzed for outliers using the nonlinear regression ROUT method with Q = 1%, the maximum desired false discovery rate. Normality of Gaussian distribution was then assessed using the D’Agnostino-Pearson ombinus K2 method. Most data were then analyzed by the non-parametric ANOVA Kruskal-Wallis test with the Mann Whitney U test for multiple comparisons of means. The data are presented as either mean (standard deviation) or as median with interquartile ranges. The ESR competitive inhibitor data were analyzed by nonlinear regression analysis. A p-value < 0.05 was considered statistically significant.

## Results

**Fig 1:**
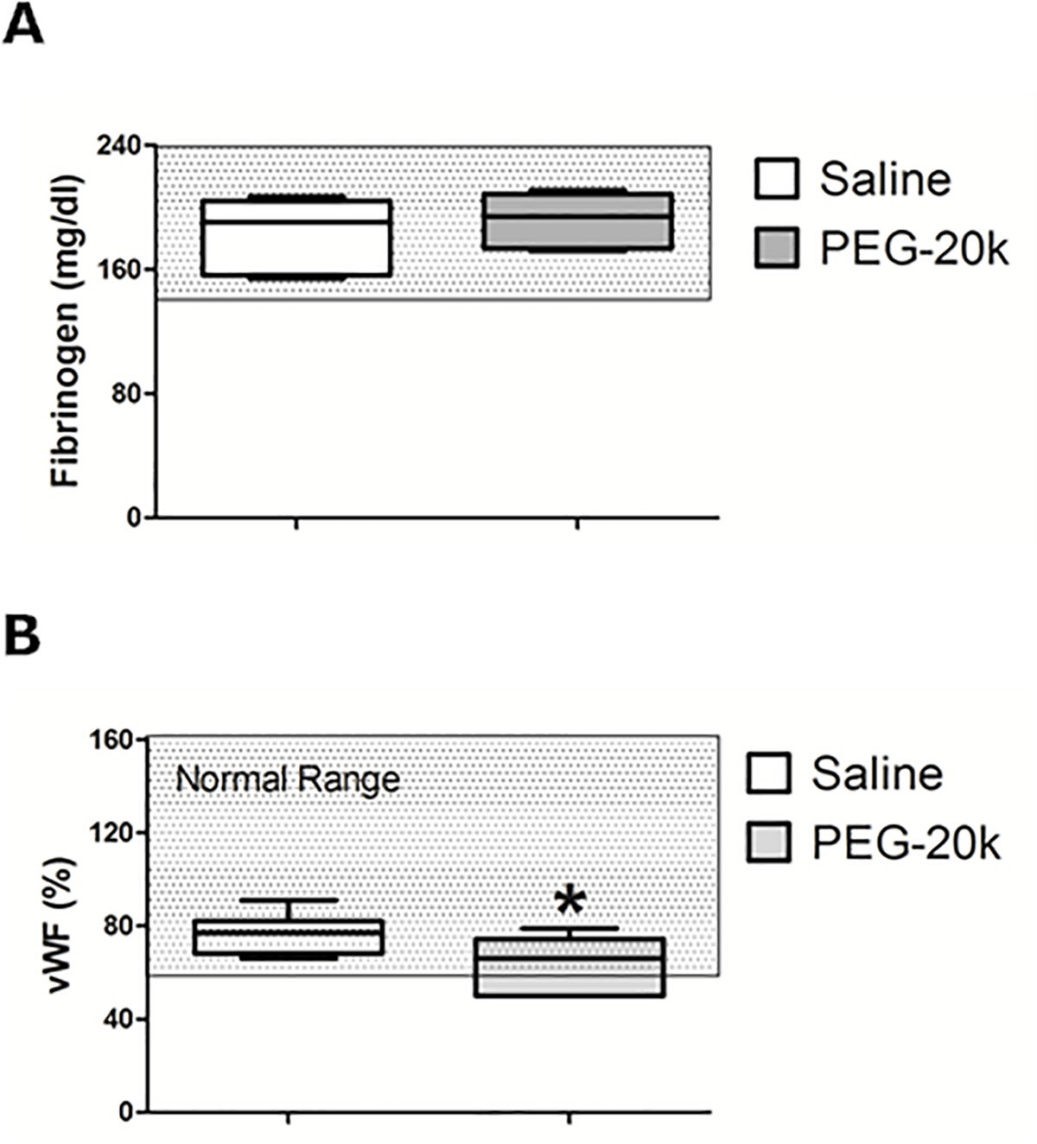
Plasma fibrinogen (panel A) and von Willebrand factor (panel B) concentrations in whole blood from healthy volunteers diluted with either saline (1 to 9 dilution) or 10% PEG-20k solution (1 to 9). The bar inside of the box is the median value of the sample, the lower and upper borders of the box represent the boundaries between the 1st and 2nd quartile and the 3rd and 4th quartile, respectively, and the ends of the whiskers indicate the population extreme values (low and high). Each group represents 6 individual values performed in triplicate. The shaded box is the normal range of values. P<0.05, relative to the saline dilutional control group [35].

The plasma concentrations of fibrinogen and von Willebrand factor (vWF) activity obtained from healthy volunteers that had been diluted 10% with either PEG-20k (10% w:v) or a saline dilution control are shown **Fig 1.** There was no difference in fibrinogen concentrations due to dilution with PEG-20k (Panel A). However, there was a small but statistically significant decrease in vWF activity observed for the PEG-20k (Panel B) samples but the levels in this group were still within the normal range.

**Fig 2:**
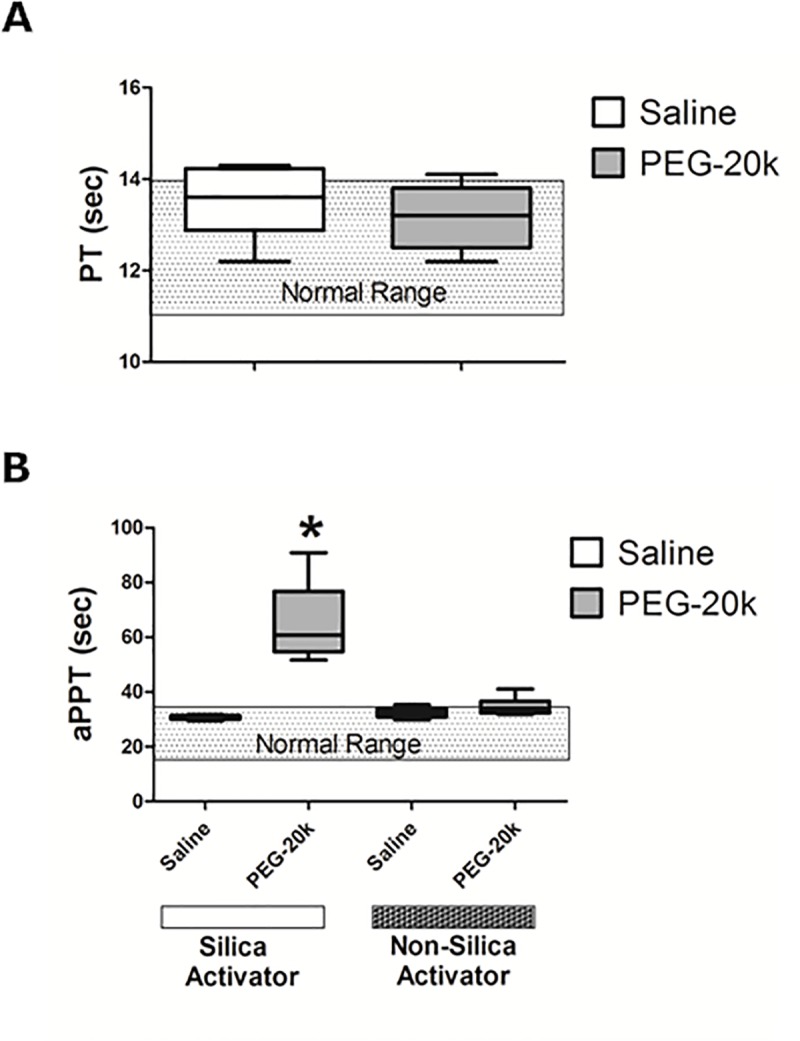
The plasma PT (panel A) and aPTT times (panel B) measured in blood from healthy volunteers diluted with either saline (1 to 9) or 10% PEG-20k solution (1 to 9). The bar inside of the box is the median value of the sample, the lower and upper borders of the box represent the boundaries between the 1st and 2nd quartile and the 3rd and 4th quartile, respectively, and the ends of the whiskers indicate the population extreme values (low and high). Each group represents 6 individual values. The shaded box is the normal range of values. P<0.05, relative to the corresponding saline dilutional control group. Panel B also shows the effects of micronized silica activator on aPTT compared to activators using kaolin.

**Fig 2** shows the PT (Panel A) and aPTT (Panel B) when plasma was diluted by 10% with either PEG-20k or saline. This is the theoretical dilution that occurs when the solutions are administered to shocked patients. These data illustrate that PEG-20k has no effect on neither PT, nor aPTT when the activator reagents included a combination of kaolin and rabbit brain phospholipids. However, when the activator for this test contained micronized silica instead of kaolin and rabbit brain phospholipids, there was a very significant prolongation in the aPTT in the PEG-20k diluted plasma samples, relative to the saline controls, suggesting that a silica-PEG-20k interaction exists that interferes with initiation of the intrinsic pathways cascade. So while PEG-20k did not have any significant effects on coagulation, it dramatically prolongs the aPTT times when micronized silica is used as an activator. This interaction has recently been observed in PEG-conjugated compounds including PEGylated factor replacement products used for patients with hemophilia [23–25]. This technical complication should be avoided when testing PEG-20k diluted blood in clinical laboratories.

**Fig 3:**
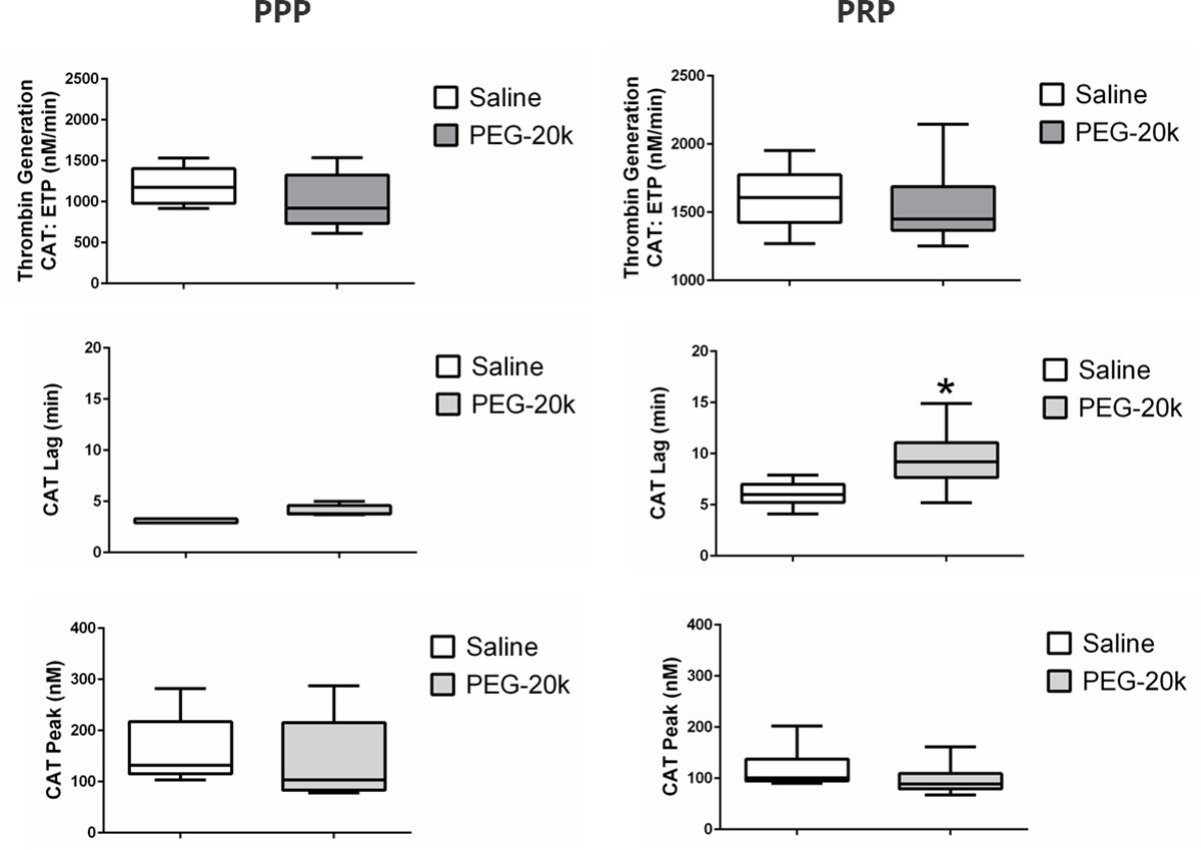
Plasma thrombin generation in PPP and PRP from healthy volunteers diluted with either saline (1 to 9) or 10% PEG-20k solution (1 to 9). CAT data shown include the ETF parameter, which is the area under the thrombin curve, the peak height of the thrombin curve, and the lag time from the time of activation until the start of thrombin generation. The bar inside of the box is the median value of the sample, the lower and upper borders of the box represent the boundaries between the 1st and 2nd quartile and the 3rd and 4th quartile, respectively, and the ends of the whiskers indicate the population extreme values (low and high). Each group represents 6 individual values. * P<0.05, relative to the saline dilutional control group.

**Fig 3** shows thrombin generation in PRP and PPP when the samples were diluted with either the PEG-20k or saline control. The presence of PEG-20k in the sample showed no effect on all CAT parameters except a slight but significantly prolonged thrombin generation lag time, but only in the PRP samples.

**Fig 4:**
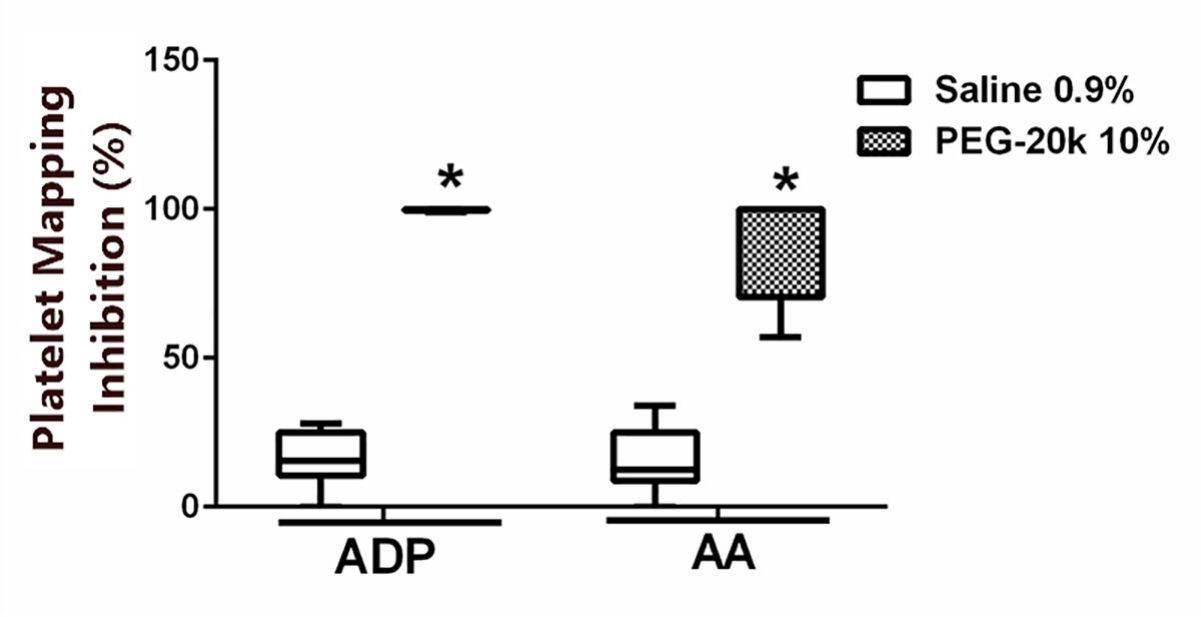
TEG platelet mapping studies conducted with whole blood obtained from volunteers diluted with either saline (1 to 9) or 10% PEG-20k solution (1 to 9). The figures shows the inhibition of activation of platelet clot formation in response to either ADP or arachidonic acid. The bar inside of the box is the median value of the sample, the lower and upper borders of the box represent the boundaries between the 1st and 2nd quartile and the 3rd and 4th quartile, respectively, and the ends of the whiskers indicate the population extreme values (low and high). Each group represents 6 individual values. P<0.05, relative to the saline dilutional control group.

The results of TEG platelet mapping, showing the platelet contribution to clot formation under platelet stimulation with either ADP or arachidonic acid (AA), is shown in **Fig 4**. Dilution with 10% PEG-20k caused a significant decrease in the ADP and AA-induced aggregation response, relative to the saline control. This is expressed as the inhibition (%) of the maximal response observed in the absence of PEG-20k or the saline vehicle.

**Fig 5:**
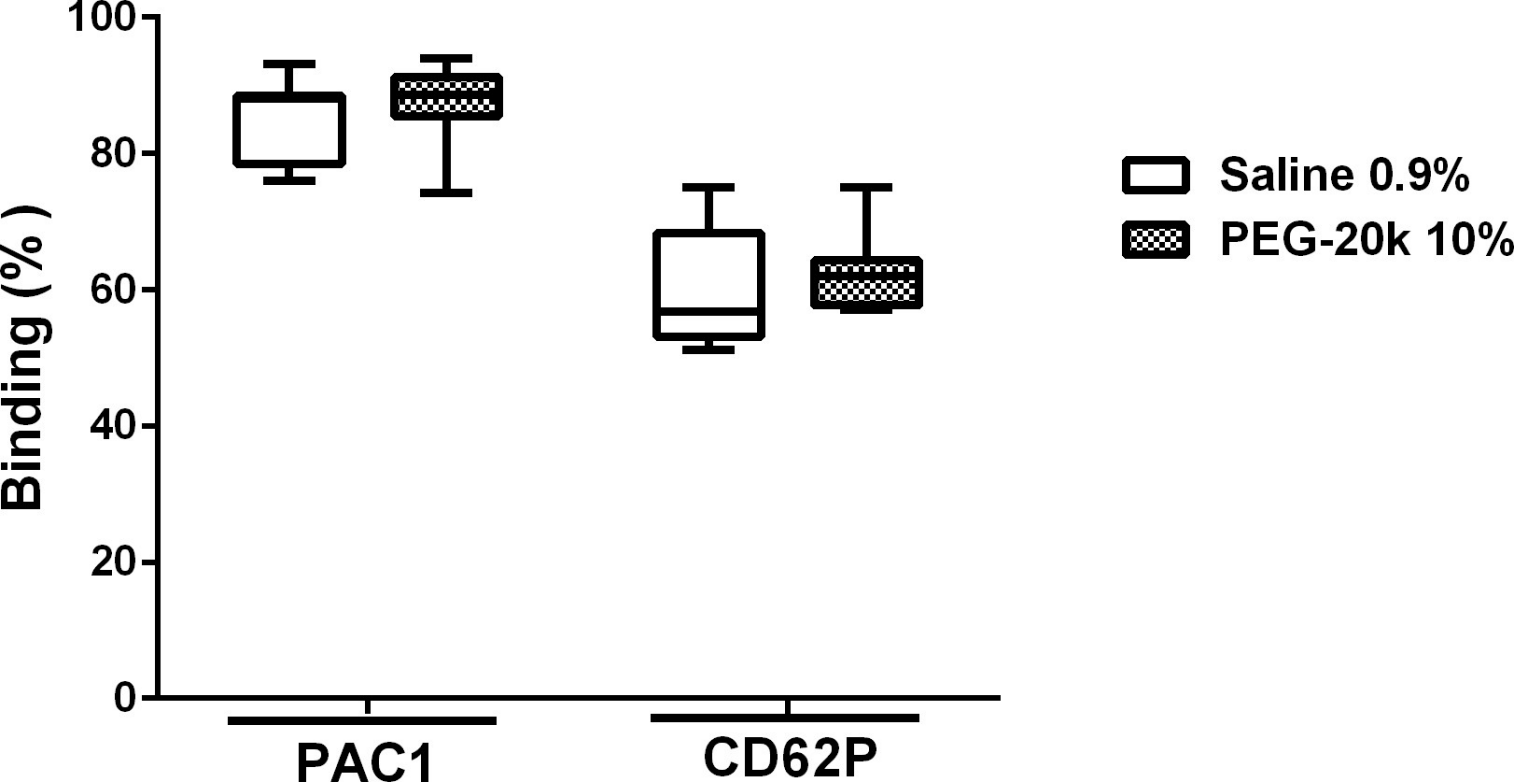
ADP-induced activation of expression of PAC1 (IIB/IIIA receptor complex) and CD62P (P-selectin) receptors on platelets in blood obtained from healthy volunteers diluted with either saline (1 to 9) or 10% PEG-20k solution (1 to 9). The bar inside of the box is the median value of the sample, the lower and upper borders of the box represent the boundaries between the 1st and 2nd quartile and the 3rd and 4th quartile, respectively, and the ends of the whiskers indicate the population extreme values (low and high). Each group represents 6 individual values.

The flow cytometry data are presented in **Fig 5**. ADP activation of platelets in PRP induces a rapid expression of glycoprotein IIb /IIIA complexes and P-Selectin that are detected by specific binding of antibodies to PAC1 and anti-CD62P, respectively. While there were significant increases in both PAC1 (88.5%) and CD62p (59.7%) antibody binding to ADP-activated platelets, compared to the non-activated state with saline dilution, the effect was not different when PEG-20k was used as the diluent (87.4% increase for PAC1 and 62.5% increase for CD62p).

**Fig 6:**
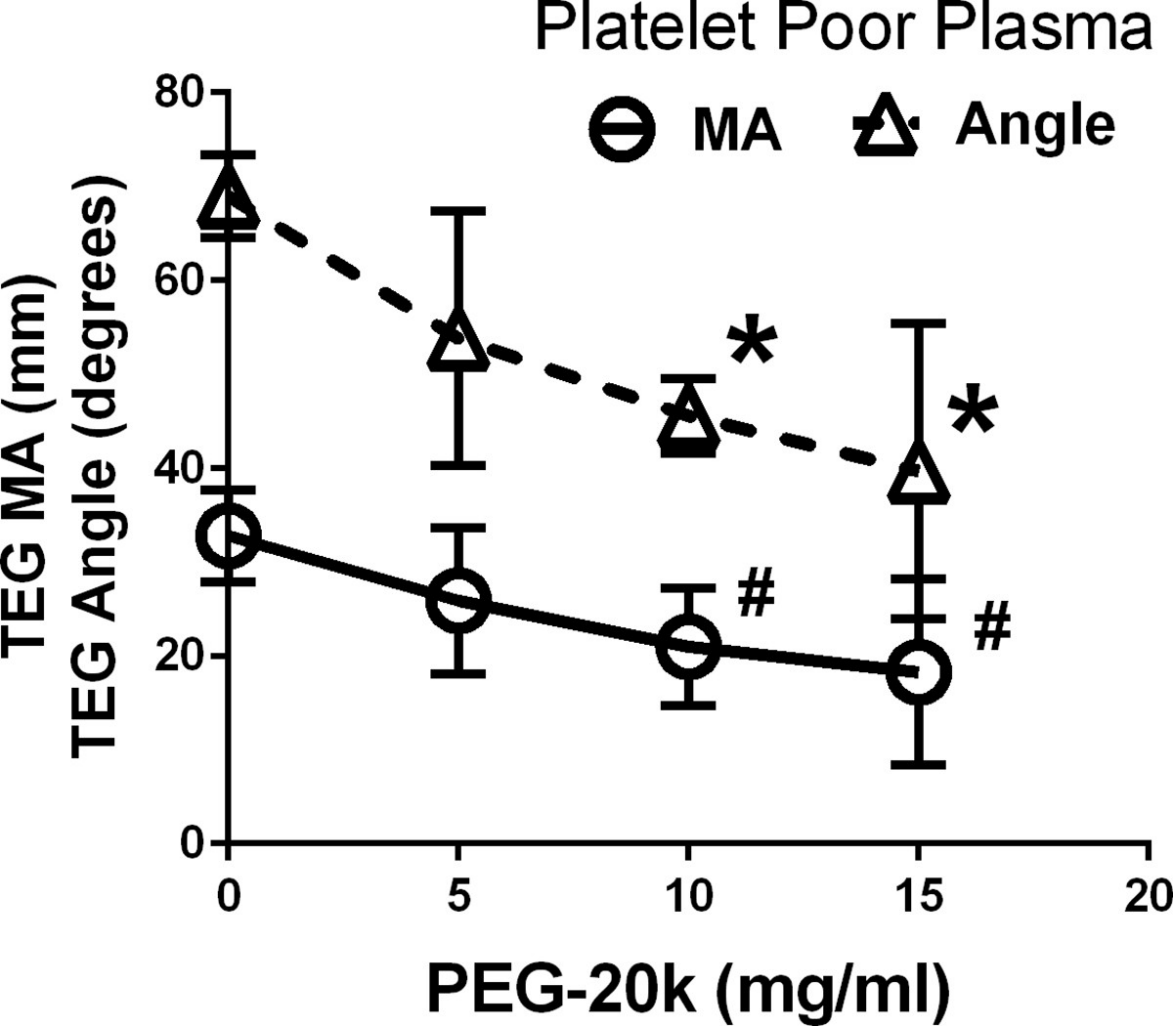
Thromboelastography values of MA and Angle from kaolin activated platelet poor plasma (PPP) obtained from healthy volunteers. All samples were diluted 10% with lactated Ringers solution containing varying concentrations of PEG-20k (0%, 5%, 10%, and 15%). Kaolin activated the PPP. Values represent mean ±SD from fresh blood obtained from 6 volunteers. * and # P<0.05, relative to the corresponding PEG-20k dilutional control values (0% PEG-20k).

The effects of PEG-20k on just the chemical phase of blood coagulation is shown in **Fig 6**. Following removal of the platelet components by centrifugation, the platelet poor plasma component was assayed in a standard TEG experiment using kaolin activation. The MA and Angle components of TEG are reported for varying doses of PEG-20k from 0–15 mg/ml with a constant volume dilution of 10% for all tests. The absolute baseline clot and kinetics are smaller compared to values obtained from volunteer whole blood [16]. The clot MA and angle were not significantly different from the dilutional control for 5 mg/ml PEG-20k but were different at the higher concentrations (P<0.05).

**Fig 7:**
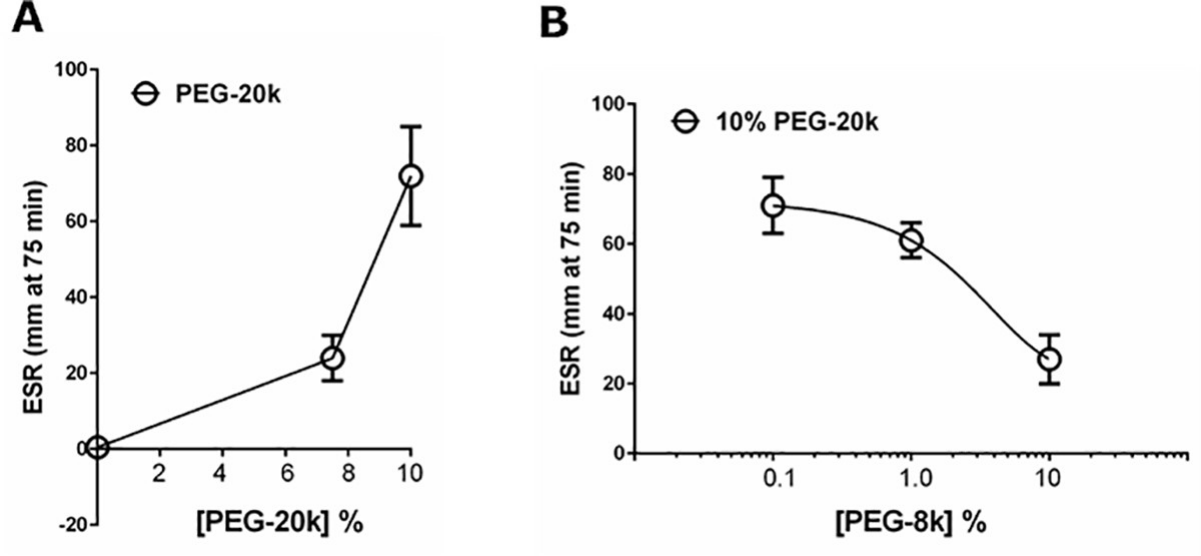
Erythrocyte Sedimentation Rates (ESR) measured in whole blood obtained from healthy volunteers diluted with saline (1 to 9) containing various concentrations of PEG-20k solution (0%, 7.5%, and 10%) (panel A). The dose dependent effects of PEG-8k on the accelerated ESR effect of 10% PEG-20k are shown in panel B. Values are mean +/- SD, n = 4 independent experiments.

Finally, in an attempt to understand the cell binding effects of PEG-20k in blood, we used the erythrocyte sedimentation rate (ESR) as a model for what may be happening in the platelet fraction with PEG-20k **(Fig 7)**. The ESR was significantly and dose-dependently increased with 7.5% and 10% PEG-20k solutions diluted 10% with whole blood (Panel A). Addition of 10% PEG-20k (weight to volume) induced a 150 fold increase in the rate of erythrocyte sedimentation, compared to the saline control at the same 10% volume dilution. In another study, the ESR sedimentation effect of 10% PEG-20k could be competitively inhibited by the addition of shorter chain PEG polymers of 1k, 4k, and 8k (8k shown in panel B).

## Discussion

PEG-20k, a new LVR crystalloid solution has recently been developed that induces profound tolerance to the low volume state when compared to other commonly used solutions. In preliminary testing using thromboelastography [16] it was determined that these solutions, which contain 10% PEG-20k, produced a dose-dependent hypocoagulative state, namely a significant decrease in MA and decreases in α-angle and k. Since MA represents clot firmness associated with platelets (80–90%) and fibrinogen (10–20%) [26], it was posited that PEG-20k effects on coagulation may interfere with platelet function and/or fibrin polymerization. Deficiencies in fibrin polymerization or fibrin cross-linking were suspected, given the decreases in the fibrinogen dependent TEG factors such as α-angle and k and changes in clot strength. These changes are affected by low fibrinogen activity, fibrinogen deficiency, Factor XIII defects, or thrombocytopenia/thrombocytopathy. Therefore, to dissect out effects of PEG on coagulation factors or platelet function, we conducted more specific testing on diluted volunteer whole blood.

This study essentially rules out PEG-20k-related effects causing coagulation protein deficiencies. For example, the concentrations of fibrinogen were not different with PEG-20k diluted blood compared to the saline controls, and the fibrinogen remained within the normal ranges. An effect of PEG-20k on fibrinogen shape or charge leading to altered catalytic rates cannot be ruled out and may be a possible avenue to explore in future studies. PEG polymers are known to camouflage active surface molecules on cells [27] so maybe the same can occur with fibrinogen molecules on platelets or in solution. Similarly, the vWF activities were slightly lower in the PEG-20k spiked plasma samples, but also remained in the normal range. Such minor changes likely cannot account for the observed effects on TEG. Therefore, the slower clot propagation and decreases in α-angle and k observed on TEG are probably not the result of lack of plasma fibrinogen or vWF activity. Similarly, since the PT and aPTT times were not different between groups, this suggests both the intrinsic and extrinsic coagulation systems are unaffected by PEG-20k. Although PEG-20k had no effects on aPTT, we observed that activator choices for this test can give a spurious effect. Specifically, the use of an activator containing micronized silica particles to start the intrinsic pathway cascade caused artifactually prolonged clotting times in the presence of PEG-20k. This PEG-silica laboratory interaction has been show previously with PEG per se [28] and with PEGylated factor replacement products [23–25]. The mechanisms for this silica effect are unknown but may be due to a preferential adherence of PEG polymers to the silica, thereby preventing its activation of factors in the intrinsic pathway. Whatever the mechanism, it is important that any future clinical aPTT testing in patients that were given PEG-20k active solutions be tested using kaolin-based activators and not micronized silica activators.

Thrombin generation is an important component of blood clotting and should be evaluated when a coagulopathy is identified since it represents the final common pathway. Furthermore, platelet dependent or independent thrombin activity may be a more important measure of coagulation than PT and aPTT times [19]. Thrombin generation, as indexed by the CAT assay, indicated a slight but significant decrease in just one measure (Lag Time) of thrombin generation and only in the PRP component of blood diluted with PEG-20k solutions, compared to the saline controls. This small change in thrombin generation was platelet dependent since it was not observed in PPP from the same blood samples. This is consistent with the other platelet-specific changes seen in this study. The contribution of this change in platelet derived thrombin activity, although statistically significant, may not represent a biologically significant factor in the observed effects of PEG-20k on TEG.

The most likely explanation for the slower clot propagation and decreased α-angle and k revolves around the axis of fibrinogen binding to activated platelets. For example, the flow cytometry data showed no difference in platelet receptor expression (PAC1 and CD62P, Fig 5) after ADP activation. However, on TEG platelet mapping, a functional analysis of platelet activation response to ADP and AA, there was a clear unresponsiveness of platelets to stimulation. This may suggest that interference by PEG-20k in platelet clot formation may be downstream from the glycoprotein IIb/IIIa receptor expression after activation. It is tempting to suggest, based on the available evidence to date, that PEG-20k may interfere with IIa/IIIb binding to fibrinogen, thereby interfering with platelet aggregation per se and the amplification of downstream receptor signaling by epinephrine, ADP, collagen, and thromboxanes on platelet aggregation. This is supported by the data showing the MA on platelet mapping and in regular TEG to be reduced with PEG-20k. Furthermore, the lower k and angle values seen with PEG-20k solutions [16], which mimic a functional state of hypofibrinogenemia in the presence of normal fibrinogen concentrations, may be due to blocking of the IIb/IIIa receptor and inhibition of fibrinogen binding and platelet aggregation. Of course, there is also the possibility that PEG-20k directly interferes with the ADP and endoperoxide receptor binding to these specific ligands. Therefore, PEG-20k may induce a state of chemical thrombasthenia at higher concentrations while not significantly affecting the coagulation cascades.

This is further supported, albeit indirectly, by data demonstrating robust effects of PEG-20k solutions on the red blood cell sedimentation rates, which are competitively inhibited by smaller PEG polymers. These data suggest that PEG-20k polymers bind to surface sites on the red blood cell to change their density, possibly through cross linking with other polymer complexes or cell components. If this were to occur in platelets too, then some platelets may be functionally removed from binding with fibrin, fibrinogen, and adhesion molecules to alter the platelet component of clot formation, as documented clearly in our previous study. This proposed parallelism between PEG-20k interactions with RBCs and platelets has not been demonstrated empirically but such a nonspecific passivation effect seems reasonable to postulate from the very strong ESR effects of PEG-20k, and from the known affinity of PEG polymers with cell membrane components, including on platelets [29–32]. Further studies using fluorescent or electron microscopy imaging may be useful to resolve any potential platelet-PEG-20k pharmacodynamic interactions under clot forming conditions. Another potential mechanism explaining PEG-20k on RBC sedimentation may involve shifts in the viscosity of the plasma phase of the blood secondary to unknown PEG-protein interactions, which alters the packing and sedimentation of the RBCs.

Although the MA value derives mostly from platelet function (80–90%), there is still a smaller component (10–20%) attributable to the fibrin component of clot formation. Since PEG-20k was seen to dose dependently interfere with MA on TEG using only platelet poor plasma, we must conclude that a smaller component of the PEG-20k effect on clot strength in clotting whole human blood is due to this fibrin component. The polymer may interfere with activated factor XIII-induced cross-linking and fXIII-fibrin interactions, thereby weakening the clot strength. This is supported by the known interference with other colloids between fXIII and fibrin to limit cross linking and weaken clot strength [33, 34]. Similarly, the platelet mapping data attributing PEG interferences with ADP and thromboxane-induced platelet aggregation and clot strength could partly be explained by a similar polymer effect on fXIII-induced cross-linking of the reptilase catalyzed fibrin that is formed under platelet mapping conditions (heparin). The exact mechanisms of how PEG polymers may cause these interferences is not known but they may involve a relatively nonspecific passivation of the polymer covering the surfaces of key binding sites on both platelets and fibrin to produce the observed effect on clot formation kinetics and strength.

In conclusion, this study has expanded our search for a mechanistic explanation for the identified effects of PEG-20k solutions on whole blood coagulation observed in healthy volunteers and trauma patients. PEG-20k seems to have less effect on the intrinsic and extrinsic coagulation pathways and on the availability of critical non-catalytic proteins such as fibrinogen and vWF. The effects of PEG-20k solutions on clot formation suggest potential interference by PEG-20k with fibrinogen binding and polymerization on the platelet thereby mimicking a state of mild functional thrombocytopenia, platelet passivation, or thrombasthenia with similar effects on fibrin cross-linking. It may be possible to chemically modify the PEG-20k functional groups to mitigate these effects.

## Supporting information

S1 File Supporting Information.pzf: Original data files and statistical analysis for all figures presented in Prism 6.0 format. (PZF)

S2 File Coags-CAT.pzf: Experimental data for the coagulation and CAT experiments, including the statistical analysis and related graphs. (PZF)

S3 File Platelet Mapping.pzfx: Experimental data for the platelet mapping experiment including the statistical analysis and the related graphics. (PZFX)

S4 File PPP TEG PEG.pzfx: Experimental data, statistical analysis, and related graphs from the thromboelastographic analysis of platelet poor plasma diluted with PEG-20k. (PZFX)

## Abbreviations

vWF: von Willebrand Factor
LVR: Low Volume Resuscitation
PEG-20k: Polyethylene Glycol 20,000 Da
NS: 0.9% NaCl
LR: Lactated Ringers
HES: Hetastarch
TEG: Thromboelastography
TICS: Trauma-Induced Capillary Leak Syndrome
PPP: Platelet Poor Plasma
ISS: Injury Severity Scor
